# It's all about balance: p53 and aging

**DOI:** 10.18632/aging.100102

**Published:** 2009-11-13

**Authors:** Benoit Biteau, Heinrich Jasper

**Affiliations:** Department of Biology, University of Rochester, Rochester, NY 14627, USA

**Keywords:** p53, aging, Drosophila, sexual dimorphism, antagonistic pleiotropy

New studies assessing whether the p53 tumor suppressor influences lifespan in flies highlight the complexities inherent in modulating the activity of this potent and versatile transcriptional regulator.

Promoting efficient regeneration while preventing cancer is critical for tissue homeostasis. Genes and processes that influence the balance between regeneration and cancer are thus likely to affect lifespan of metazoans. Supporting this view, the tumor suppressor p53 has been found to strongly influence aging in mice. Interestingly, the lifespan consequences of increasing or decreasing p53 activity in mice are complex [1-5]. It is no surprise that loss of p53 leads to increased cancer incidence, and thus shorter lifespan [2]. Yet increasing p53 activity can have pleiotropic consequences: such interventions generally prevent cancer, increasing lifespan in some cases, but can also cause accelerated aging in others [6-11]. The discrepancy between lifespan shortening and lifespan extending consequences of p53 gain-of-function conditions has been attributed to differences in regulation of the corresponding transgenes, and highlights the complex and dose-dependent effects that such a versatile regulator of cell proliferation, repair and death can have on health span and aging [2]. The exact reasons for the pleiotropic effects of p53 on aging, however, remain elusive.

Aging studies in less complex model organisms mightbe expected to contribute important  insights into this puzzle. New studies by the Tower and Helfand labs  assess the consequences of modulating p53 activity for the lifespan of flies, and shed light on the complexities of p53 function in even such relatively simple organisms [12-15].

In an exhaustive analysis of the lifespan effects of p53 gain- and loss-of-function conditions, Waskar and colleagues find strikingly pleiotropic effects resulting from ubiquitous increase or decrease of wild type p53 function [12]. Importantly, the consequences of modulating p53 are found to be tissue-, stage- and sex-specific: ubiquitous over-expression of p53 in adults shortens lifespan in females but slightly increases lifespan in males [12], whereas neuronal expression of the same construct extends lifespan in females and decreases life span in males [13]. Over-expressing p53 in larval stages, on the other hand, is sufficient to extend adult lifespan in both sexes, but in a dose-dependent manner, where strong expression is deleterious for lifespan, while moderate to weak over-expression increases lifespan [12].

The authors further examine the lifespan of a battery of mutants in which the endogenous p53 gene is disrupted, and find robust increase of lifespan in females, but context-dependent effects in males [12]. Confirming earlier studies by the Helfand lab, which demonstrated lifespan extension when dominant-negative p53 is expressed in neurons [16-18], Waskar and colleagues find that ubiquitous expression of a dominant negative form of p53 extends lifespan moderately in females [12].

The pleiotropic and sex-specific effects of modulating p53 activity in adults are likely caused by specific functions of this tumor suppressor in particular bio-logical processes. It is tempting to speculate that p53 might act to compromise or maintain the function of various tissues and processes in specific ways, thus causing antagonistic effects on overall lifespan. For example, (i) p53 is expressed in the female germline [19,20], and modulating p53 expression in this tissue is likely to affect its function. The ablation of germline stem cells has recently been shown to cause robust lifespan extension in *Drosophila*[21], suggesting that the effects of ubiquitous over-expression of p53 might in part be mediated by p53 function in this tissue.

(ii) expression of p53 in intestinal stem cells (ISCs) leads to cell loss and strongly impairs gut regeneration [22]. Interestingly, probably due to a higher nutrient demand, intestinal tissue turnover is faster in females than in males [22]. As a consequence, p53-induced loss of ISCs would negatively affect female lifespan preferentially, counteracting the beneficial effects of neuronal expression or reduced germline function.

(iii) the regulation of insulin-like peptide production by p53 (described by [16] is likely to influence tissue function systemically and thus further complicate the effects of p53 on lifespan. Accordingly, Foxo modulates the sex-specific lifespan consequences of p53 over-expression [13].

(iv) the lifespan extending effects of a dominant mutant p53 transgene were not additive with Sir2 over-expression or dietary restriction (DR) [14,15], and the life span extension due to wild-type p53 over-expression was dependent upon Sir2 function [13], indicating that tissue-specific interactions between these proteins might control metabolic homeostasis, further complicating the lifespan effects of modulating p53 activity.

The fly offers unique tools to start testing these hypotheses, and it can thus be expected that the tissue-specific functions of p53, and the relative contribution of these functions to overall lifespan will be further explored in the near future.  Such studies are expected to help obtaining a comprehensive view of the effects of p53 on tissue homeostasis, metabolic control, and other physiological processes that influence aging.
